# Birth Weight and Prenatal Exposure to Polychlorinated Biphenyls (PCBs) and Dichlorodiphenyldichloroethylene (DDE): A Meta-analysis within 12 European Birth Cohorts

**DOI:** 10.1289/ehp.1103767

**Published:** 2011-10-13

**Authors:** Eva Govarts, Mark Nieuwenhuijsen, Greet Schoeters, Ferran Ballester, Karolien Bloemen, Michiel de Boer, Cécile Chevrier, Merete Eggesbø, Mònica Guxens, Ursula Krämer, Juliette Legler, David Martínez, Lubica Palkovicova, Evridiki Patelarou, Ulrich Ranft, Arja Rautio, Maria Skaalum Petersen, Rémy Slama, Hein Stigum, Gunnar Toft, Tomas Trnovec, Stéphanie Vandentorren, Pál Weihe, Nynke Weisglas Kuperus, Michael Wilhelm, Jürgen Wittsiepe, Jens Peter Bonde, and OBELIX/ENRIECO

**Affiliations:** 1 Environmental Risk and Health, Flemish Institute for Technological Research (VITO), Mol, Belgium; 2 CIBER de Epidemiología y Salud Pública (CIBERESP), Spain; 3 Center for Research in Environmental Epidemiology (CREAL), Barcelona, Spain; 4 Hospital del Mar Research Institute (IMIM), Barcelona, Spain; 5 Department of Biomedical Sciences, University of Antwerp (UA), Antwerp, Belgium; 6 Centre of Public Health Research (CSISP), Valencia, Spain; 7 School of Nursing, University of Valencia, Valencia, Spain; 8 Department of Health Sciences, VU University, Amsterdam, the Netherlands; 9 Inserm, Rennes, France; 10 University of Rennes I, Rennes, France; 11 Department of Genes and Environment, Norwegian Institute of Public Health, Oslo, Norway; 12 IUF – Leibniz Research Institute for Environmental Medicine, Duesseldorf, Germany; 13 Institute for Environmental Studies, VU University, Amsterdam, the Netherlands; 14 Slovak Medical University, Bratislava, Slovakia; 15 Department of Social Medicine, University of Crete, Heraklion, Greece; 16 Centre for Arctic Medicine, Thule Institute, University of Oulu, Oulu, Finland; 17 Department of Occupational Medicine and Public Health, Faroese Hospital System, Faroe Island; 18 Inserm, Team of Environmental Epidemiology applied to Reproduction and Respiratory Health, Grenoble, France; 19 Grenoble University, Institut Albert Bonniot, Grenoble, France; 20 Department of Occupational Medicine, Aarhus University Hospital, Aarhus, Denmark; 21 National Institute of Public Health Surveillance, Saint Maurice, France; 22 Department of Pediatrics, Erasmus Medical Center – Sophia Children’s Hospital, Rotterdam, the Netherlands; 23 Department of Hygiene, Social and Environmental Medicine, Ruhr-University Bochum, Bochum, Germany; 24 Department of Occupational and Environmental Medicine, Copenhagen University Hospital Bispebjerg, Copenhagen, Denmark

**Keywords:** birth cohort studies, DDE, gestational age, meta-analysis, PCBs, reproductive effects

## Abstract

Objectives: Exposure to high concentrations of persistent organochlorines may cause fetal toxicity, but the evidence at low exposure levels is limited. Large studies with substantial exposure contrasts and appropriate exposure assessment are warranted. Within the framework of the EU (European Union) ENRIECO (ENvironmental Health RIsks in European Birth Cohorts) and EU OBELIX (OBesogenic Endocrine disrupting chemicals: LInking prenatal eXposure to the development of obesity later in life) projects, we examined the hypothesis that the combination of polychlorinated biphenyls (PCBs) and dichlorodiphenyldichloroethylene (DDE) adversely affects birth weight.

Methods: We used maternal and cord blood and breast milk samples of 7,990 women enrolled in 15 study populations from 12 European birth cohorts from 1990 through 2008. Using identical variable definitions, we performed for each cohort linear regression of birth weight on estimates of cord serum concentration of PCB-153 and *p*,*p*´-DDE adjusted for gestational age and *a priori* selected covariates. We obtained summary estimates by meta-analysis and performed analyses of interactions.

Results: The median concentration of cord serum PCB-153 was 140 ng/L (range of cohort medians 20–484 ng/L) and that of *p*,*p*´-DDE was 528 ng/L (range of cohort medians 50–1,208 ng/L). Birth weight decreased with increasing cord serum concentration of PCB-153 after adjustment for potential confounders in 12 of 15 study populations. The meta-analysis including all cohorts indicated a birth weight decline of 150 g [95% confidence interval (CI): –250, –50 g] per 1-µg/L increase in PCB-153, an exposure contrast that is close to the range of exposures across the cohorts. A 1-µg/L increase in *p*,*p*´-DDE was associated with a 7-g decrease in birth weight (95% CI: –18, 4 g).

Conclusions: The findings suggest that low-level exposure to PCB (or correlated exposures) impairs fetal growth, but that exposure to *p*,*p*´-DDE does not. The study adds to mounting evidence that low-level exposure to PCBs is inversely associated with fetal growth.

From the 1930s, polychlorinated biphenyls (PCBs) have been manufactured in large quantities for use in many industrial applications until they were banned in most countries in the 1970s. Dichlorodiphenyltrichloroethane (DDT) is a pesticide that since the 1940s has been used worldwide for malaria vector control, and it is still in use in some areas (Aneck-Hahn et al. 2007; Ayotte et al. 2001). PCBs and the main DDT metabolite dichlorodiphenyldichloroethylene (*p*,*p*´-DDE) bioaccumulate in fat tissues, biomagnify through the food chain, are highly persistent in living organisms, and comprise the bulk of organochlorine residues in human tissues (Longnecker et al. 2003). The concentrations of these chemicals in blood, fat, and milk have decreased over the past 30 years but are still detectable in blood of the general population all over the globe (Jonsson et al. 2005). It has been suggested that organochlorines may interfere with fetal growth through interaction with endogenous steroid hormone signaling (Faroon and Olson 2000; Lopez-Espinosa et al. 2009). Birth weight restriction related to PCBs was first described in humans after the 1968 Yusho incident in Japan, where thousands of pregnant women were intoxicated by PCB-contaminated cooking rice oil (Yamashita and Hayashi 1985). During the past 15 years, additional research has addressed human reproductive toxicity of PCBs in studies of fishing communities, fish eaters, and the general population in various regions, as reviewed by Toft et al. (2004) and Longnecker et al. (2003). Findings from these studies on the relation between maternal PCB exposure and birth weight are not consistent—some indicating an inverse association (Fein et al. 1984; Karmaus and Zhu 2004; Murphy et al. 2010; Patandin et al. 1998; Rylander et al. 1995, 1996, 1998; Wojtyniak et al. 2010), an inverse association among male infants only (Sonneborn et al. 2008), a positive association (Dar et al. 1992), or no association at all (Gladen et al. 2003; Grandjean et al. 2001; Longnecker et al. 2005; Mendez et al. 2010; Vartiainen et al. 1998).

A large U.S. study of women giving birth between 1959 and 1966 (when DDT was still being used) found a dose–response relationship between DDE concentration in maternal serum and low birth weight (Longnecker et al. 2001). Children in the high exposure group (> 60 ng/g) weighed 150 g less and were born about 1 week earlier than the children in the low exposure group (< 15 ng/g). Similar results were observed in an Indian study of pregnant women with high DDT exposures (Siddiqui et al. 2003), but serum DDT and/or DDE was not associated with birth weight in two other studies (Gladen et al. 2003; Wojtyniak et al. 2010).

Many factors may contribute to the discrepant findings in earlier observational studies of reproductive effects of PCBs. This includes correlations in some populations between persistent organochlorines (POCs) and such dietary nutrients as n-3 fatty acids that may increase gestational age (Grandjean et al. 2001), limited study size, insufficient exposure levels or contrasts between compared populations (Wojtyniak et al. 2010), exposure to different mixtures of PCB congeners (Murphy et al. 2010), confounding by other organochlorines such as hexachlorobenzene (HCB) (Eggesbo et al. 2009), and different susceptibility of studied populations (Sonneborn et al. 2008). Publication bias could also be a problem, with studies not finding an association being underreported.

A recent review concluded that large-scale studies with a sufficient number of participants in well-defined groups with substantial exposure contrast are needed to further elucidate the possible adverse effects of POCs on human reproductive health (Toft et al. 2004). The ENRIECO (ENvironmental Health RIsks in European birth COhorts) framework of European birth cohorts and the cohorts included in the EU (European Union) OBELIX (OBesogenic Endocrine disrupting chemicals: LInking prenatal eXposure to the development of obesity later in life) project provide the basis for such a study.

The objective of this study was to examine associations between biological markers of exposure to POCs and birth weight in 12 European birth cohorts, including possible modifying effects of sex and tobacco smoking.

## Methods

*Description of the cohorts.* The ENRIECO project has provided an inventory of European birth cohorts that aim to investigate the impact of environmental exposures on health in pregnancy and early childhood. Among these cohorts, we identified 14 cohorts of pregnant women with individual measurements of PCB-153 and *p*,*p*´-DDE. One invited cohort declined participation for reasons not related to current hypotheses, and we excluded one cohort that used placenta tissue samples for exposure assessment [the Finnish LUKAS cohort (Karvonen et al. 2009)]. Each cohort targeted the general population and included births from 1990 to 2008. We divided the INMA (INfancia y Medio Ambiente; Environment and Childhood) cohort into two populations based upon the matrix used for POC measurements (maternal serum or cord serum) and divided the INUENDO cohort into three populations (Greenland; Warsaw, Poland; and Kharkiv, Ukraine) resulting in 15 study populations with a total of 7,990 live-born singletons with PCB-153 measurements and 7,788 live-born singletons with *p*,*p*´-DDE measurements (Table 1). The concentrations of PCBs and *p*,*p*´-DDE were analyzed in maternal serum or whole blood collected during pregnancy (seven populations), cord serum or plasma (five populations), or breast milk (three populations). Detailed information regarding the study cohorts are provided in Table 1 and Supplemental Material, Table 1 (http://dx.doi.org/10.1289/ehp.1103767).

**Table 1:** Description of the ENRIECO/OBELIX birth cohorts with biological PCB‑153/*p*,*p*´-DDE exposure biomarkers included in the present study.

Cohort	Setting location	Time period	Enrollment method	Only babies at term?	Other exclusion criteria	Participation rate	Exposure assessment			Main reference
							Selection criteria for exposure assessment	*na*		
								Biological matrix	Time of collection		PCB-153	*p*,*p*´- DDE
GRD	The Netherlands (Groningen–Rotterdam)	1990–1992	During prenatal consultations in late pregnancy by obstetricians or midwifes	Yes	Serious illness during pregnancy; congenital anomalies; white race; parity > 2; cesarean section	70%		Availability of biological samples	Cord plasma	At birth		382	—		Huisman et al. 1995
	Germany (Düsseldorf)	1993–1995	At delivery from the obstetrical wards of three Düsseldorf hospitals by three medical students	Yes	Serious illness during pregnancy; congenital anomalies; native German families; parity > 2; cesarean section	70%		Availability of biological samples	Cord serum	At birth		141	—		Walkowiak et al. 2001
FAROES2 (cohort 2)	Faroe Islands	1994–1995	Consecutive births at the National Hospital in Torshavn, but from women living away from the capital area of Torshavn	No	Serious congenital disease	64%		Availability of biological samples	Maternal serum	Week 34 (from antenatal consultation)		173	173		Steuerwald et al. 2000
FAROES3 (cohort 3)	Faroe Islands	1997–2000	Consecutive pregnant women	No	Serious congenital disease	60%		Breast-feeding	Breast milk	Days 3–5 and at 2 weeks		596	596		Weihe et al. 2003
INMA	Spain (Menorca)	1997–1999	During prenatal care at general practices of the island (in public or private health centers)	No	Maternal age < 16 years; to have followed any program of assisted reproduction; no wish to deliver in the reference hospital; speaking difficulties	98%		Availability of biological samples	Cord serum	At birth		404	405		Carrizo et al. 2006
	Spain (Granada)	2000–2002	During hospital admission for delivery in the study area	No	Maternal age < 16 years; to have followed any program of assisted reproduction; no wish to deliver in the reference hospital; speaking difficulties	Unknown		Availability of biological samples	Cord serum	At birth		—	318		Ribas-Fito et al. 2006
	Spain (Valencia)	2004–2005	During the first prenatal visit in the main public hospital or health center of the study area	No	Maternal age < 16 years; to have followed any program of assisted reproduction; no wish to deliver in the reference hospital; speaking difficulties	54%		Availability of biological samples	Cord serum	At birth		499	499		Vizcaino et al. 2010
	Spain (Sabadell)	2004–2006	During the first prenatal visit in the main public hospital or health center of the study area	No	Maternal age < 16 years; to have followed any program of assisted reproduction; no wish to deliver in the reference hospital; speaking difficulties	60%		Availability of biological samples	Maternal serum	Week 13 of pregnancy		605	605		Ribas-Fito et al. 2006
	Spain (Asturias)	2004–2007	During the first prenatal visit in the main public hospital or health center of the study area	No	Maternal age < 16 years; to have followed any program of assisted reproduction; no wish to deliver in the reference hospital; speaking difficulties	45%		Availability of biological samples	Cord serum	At birth		25	25		Ribas-Fito et al. 2006
	Spain (Gipuzkoa)	2006–2008	During the first prenatal visit in the main public hospital or health center of the study area	No	Maternal age < 16 years; to have followed any program of assisted reproduction; no wish to deliver in the reference hospital; speaking difficulties	68%		Availability of biological samples	Maternal and cord serum	Week 13.5 of pregnancy and at birth		604	605		Ribas-Fito et al. 2006
DUISBURG	Germany (Duisburg)	2000–2002	Self-selected pregnant women within a predefined area mainly in Duisburg South	Yes	Nonhealthy mother–infant pairs; babies not from German- or Turkish-speaking families; Apgar score < 8; parity > 3	Unknown		Availability of biological samples	Maternal blood	32nd week of pregnancy		189	189		Wilhelm et al. 2008; Wittsiepe et al. 2008
*continued next page*															
Table 1. continued.															
Cohort	Setting location	Time period	Enrollment method	Only babies at term?	Other exclusion criteria	Participation rate		Exposure assessment							Main reference
								Selection criteria for exposure assessment				*na*			
									Biological matrix	Time of collection		PCB-153	*p*,*p*´- DDE		
FLEHSI	Belgium (Flanders)	2002–2004	At delivery in maternities of eight districts covering 20% of Flanders’ area	No	Complications in delivery; living < 5 years in the area; not Dutch reading	98%		Availability of biological samples	Cord plasma	At birth		1,068	1,114		Koppen et al. 2009
INUENDO	Greenland	2002–2004	By the local midwife when visiting the local hospital or health clinic from 15 municipalities of all regions in Greenland	No	Maternal age < 18 years; not born in the country	90%		Availability of biological samples	Maternal serum	24 weeks on average		546	546		Toft et al. 2005
	Poland (Warsaw)	2003–2004	During antenatal classes at the obstetric outpatient clinic of the Gynaecological and Obstetric Hospital of the Warsaw School of Medicine or with physicians at a collaborating hospital in the same city	No	Maternal age < 18 years; not born in the country	68%		Availability of biological samples	Maternal serum	33 weeks on average		199	199		Toft et al. 2005
	Ukraine (Kharkiv)	2003–2004	During visit of one of eight antenatal clinics or three maternity hospitals in Kharkiv by gynecologists	No	Maternal age < 18 years; not born in the country	26%		Availability of biological samples	Maternal serum	24 weeks on average		589	589		Toft et al. 2005
Michalovce	Slovakia	2002–2004	At delivery in maternities of two districts, one with high contamination of PCBs (Michalovce), and another one upwind and upstream of the chemical facility with lower contamination levels (Svidnik).	No	Mothers with major illness; severe congenital anomalies; maternal age < 18 years; living < 5 years in the area; parity > 4	60%		Availability of biological samples	Cord serum	At birth		1,082	1,082		Hertz-Picciotto et al. 2003
HUMIS	Norway	2002–2006	2–4 weeks after birth during the routine health visit at home	No	Non-fluent in Norwegian	64%		Random selection in the cohort; breast-feeding	Breast milk	Milk sampled on 8 consecutive days and pooled		418	418		Eggesbo et al. 2009
PELAGIE	France (Brittany)	2002–2006	During first prenatal visit by gynecologists or obstetricians in the study area	No	Inclusion later than 19 weeks of pregnancy	80%		Stratified random selection of a subcohort among the live born cohort; availability of biological samples	Cord serum	At birth		396	395		Petit et al. 2010
ELFE pilot	France	2007	At delivery in maternity department	No	Maternal age < 18 years; not French speaking; parity > 2	55%		Breast-feeding	Breast milk	1 month after birth		44	—		Vandentorren et al. 2009
RHEA	Greece (Heraklion, Crete)	2007–2008	Contact by interviewer of all pregnant women living in Heraklion around 12 weeks of gestation	No	Maternal age < 18 years; insufficient understanding of the Greek language	72%		Random selection of a small subcohort	Maternal serum	During the first interview		30	30		Vardavas et al. 2010
**a**Number of liveborn singleton births with exposure levels.															

*Exposure assessment.* Analysis of PCB-153 and *p,p*´-DDE. We used PCB-153 as a biomarker of PCB exposure because concentrations of this PCB congener are relatively high and highly correlated with the total molar concentration of PCBs (Glynn et al. 2000; Muckle et al. 2001; Needham et al. 2011). The elimination half-life of PCB-153 is > 10 years (Ritter et al. 2011 and that of *p*,*p*´-DDE, the major metabolite of DDT, is approximately 5 years (Ferreira et al. 2011).

To facilitate comparisons of results from cohorts using different matrices for exposure assessment, we expressed all contaminant levels as wet weight cord serum levels, which directly reflect fetal exposure at the time of delivery (some compounds do not cross the placenta efficiently). For populations that did not have cord blood analyses, we estimated concentrations in cord serum from the concentrations measured in maternal serum (FAROES2, INMA, INUENDO, and RHEA), maternal whole blood (DUISBURG), or breast milk [FAROES3, HUMIS (Norwegian Human Milk Study), and ELFE (Growing up in France) pilot], using the following conversion factors [for details, see Supplemental Material, pp. 2–3 (http://dx.doi.org/10.1289/ehp.1103767)]:

Cord serum level (ng/L) = 0.20 × maternal serum level (ng/L)

Cord serum level (ng/L) = 1.20 × breast milk level (ng/g fat)

Cord serum level (ng/L) = 0.36 × maternal whole blood level (ng/L).

We compiled information about chemical-analytical methods and their detection limits [see Supplemental Material, Table 2 (http://dx.doi.org/10.1289/ehp.1103767)]. High-quality analytical data were available for both PCB-153 and *p*,*p*´-DDE in each cohort except the GRD (Groningen–Rotterdam–Düsseldorf) and ELFE pilot cohorts, where only PCB-153 was measured. Spearman correlation coefficients between PCB-153 and *p*,*p*´-DDE in the cohorts ranged from 0.3 to almost 1, with a median of 0.6.

**Table 2:** Concentration of PCB-153 and *p*,*p*´-DDE (ng/L) exposure biomarkers in cord serum, actual or obtained by conversion, of the ENRIECO/OBELIX birth cohorts.

PCB-153 (ng/L)							*p*,*p*´-DDE (ng/L)						
Cohort	*n*	Mean ± SD	Median	*n* < LOD/LOQ	*n*	Mean ± SD	Median	*n* < LOD/LOQ
GRD*a*		523		170.6 ± 99.7		150.0		0		—		—		—		—
FAROES2*b*		167		648.4 ± 580.0		484.2		2 (1.2%)		167		1708.3 ± 1433.9		1208.0		0
FAROES3*c*		549		434.4 ± 347.6		345.6		0		549		987.7 ± 994.9		729.6		0
INMA cord*a*		1,227		156.1 ± 112.7		134.1		114 (9.3%)		1,515		1,380 ± 2269.8		596.0		57 (3.8%)
INMA mat*b*		856		52.8 ± 34.7		46.4		56 (6.5%)		857		262.6 ± 1030.0		131.2		5 (0.6%)
DUISBURG*b*		189		147.1 ± 99.4		124.0		0		189		323.7 ± 435.6		216.0		0
FLEHSI*a*		1,015		73.3 ± 56.7		60.0		202 (19.9%)		1,061		315.6 ± 344.6		220.0		19 (1.8%)
Greenland*b*		546		253.8 ± 341.9		155.1		7 (1.3%)		546		634.6 ± 684.2		435.7		10 (1.8%)
Warsaw*b*		199		23.8 ± 17.8		20.1		43 (21.6%)		199		824.7 ± 518.3		668.8		0
Kharkiv*b*		577		45.8 ± 36.3		37.1		20 (3.5%)		577		1100.8 ± 762.3		916.3		0
Michalovce*a*		1,036		393.5 ± 458.3		271.8		2 (0.2%)		1,036		1329.5 ± 1338.4		1014.7		8 (0.8%)
HUMIS*c*		409		43.1 ± 20.0		39.2		0		409		75.0 ± 111.0		49.8		0
PELAGIE*a*		396		126.1 ± 77.7		110.0		2 (0.5%)		395		253.3 ± 335.8		180.0		76 (19.2%)
ELFE pilot*c*		43		100.4 ± 52.7		92.5		0		—		—		—		—
RHEA*b*		30		28.0 ± 13.4		23.8		0		30		573.2 ± 389.2		496.0		0
Combined		7,762		179.8 ± 156.6		139.6		448 (5.8%)		7,530		751.5 ± 819.1		527.9		175 (2.3%)
mat, maternal serum. **a**Observed cord serum concentrations and **b**estimated cord serum concentrations based on measured concentrations in maternal serum (whole blood for DUISBURG) or **c**breast milk [for additional information on conversions, see Supplemental Material, pp. 2–3 (http://dx.doi.org/10.1289/ehp.1103767)].																

*Outcome variable and covariates/confounders.* The outcome of interest was birth weight, which was extracted from medical records. Covariate data were obtained from questionnaires and medical record information for children and their mothers, including information from antenatal health care visits. Gestational age was estimated from the date of the last menstrual period and/or by ultrasound. For five study populations, the data obtained from the last menstrual period were replaced by ultrasound determination if the discrepancy between the two techniques exceeded 7–14 days [see Supplemental Material, Table 1 (http://dx.doi.org/10.1289/ehp.1103767)]. All participants completed an extensive questionnaire themselves or by a telephone or face-to-face interview, assessing information on lifestyle, diet, use of tobacco and alcohol, residence history, health, education, hobbies, and occupation (if applicable). Each study was approved by appropriate national ethical committees, and mothers provided written informed consent for participation.

*Statistical analysis.* We developed a uniform script in SAS version 9.1 (SAS Institute Inc., Cary, NC, USA) and SPSS versions 17.0 and 18.0 (SPSS, Chicago, IL, USA) to define the variables, units, and categories used in statistical analyses for each of the 11 study centers. We allowed cohort specific variables for region and socioeconomic status. For some important confounders, missing values were replaced by the most frequent outcome for the study population. Specifically, missing data for smoking and alcohol consumption were replaced by nonsmoking/nondrinker, except for (in the INUENDO cohort) Greenland, where mothers with missing data for smoking were assumed to be smokers, and Warsaw, where mothers with missing data for alcohol were assumed to be moderate drinkers. For continuous variables (e.g., maternal height) missing values were replaced with the population-specific median.

In all 15 study populations, PCB-153 and *p*,*p*´-DDE were detected in at least 78% of subjects, and in 11 populations both compounds were detected in > 95% of samples (Table 2). We replaced values below the limit of detection (LOD) or quantification (LOQ) by the LOD or LOQ divided by the square root of two (Hornung and Reed 1990).

We developed separate multiple linear regression models to estimate the association between birth weight and PCB-153 or *p*,*p*´-DDE concentrations in cord serum. Several known determinants of birth weight were included in the models regardless of actual degree of confounding of the POC–birth weight association in the individual cohorts (Bailey and Byrom 2007; Goldenberg et al. 1997). These included gestational age (weeks, linear and quadratic terms), offspring sex (male/female), region (cohort-specific categories), maternal body mass index (BMI; < 18.5 kg/m², 18.5 ≤ 25 kg/m², 25 ≤ 30 kg/m², and ≥ 30 kg/m²), height (continuous), smoking status during pregnancy (non-smoking, smoking ≤ 9 cigarettes/day, smoking > 9 cigarettes/day), socioeconomic status (cohort-specific categories), mother’s age (< 25 years, 25–29 years, 30–34 years, and ≥ 35 years), parity (0, 1, and ≥ 2), ethnicity (cohort-specific categories), and gestational age at time of sampling (first, second, and third trimester and postnatal). We evaluated sex and smoking status as potential effect modifiers based on literature regarding PCB exposure and growth or development (Hertz-Picciotto et al. 2005; Verhulst et al. 2009), and we also examined interaction between PCB-153 and *p*,*p*´-DDE. Effect modification (interaction) was analyzed in models including main effects and cross-product terms. A *p*-value < 0.05 for the effect of the cross-product was taken as an indication of interaction. Finally, we examined associations of gestational age with PCB-153 and *p*,*p*´-DDE in cord serum by linear regression to determine if associations with birth weight might be mediated by effects on gestational age.

We checked assumptions of normality, constancy of variance, independence (randomness), and linearity with informal diagnostic plots and formal tests [White’s General test for constancy of variance (White 1980), Kolmogorov–Smirnov test for normality, and the lack of fit test for linearity (Neter et al. 1996)] and fitted regression models with and without influential outliers.

We used meta-analyses to estimate the overall summary effects of levels of PCB-153 and *p*,*p*´-DDE in cord serum on birth weight using R version 2.11.0 (R Foundation for Statistical Computing, Vienna, Austria). First, we tested for heterogeneity between effect estimates using the *Q*-test (Cochran 1954). If the result of the *Q*-test was statistically significant (*p* < 0.05), an indication of heterogeneity across populations, we used random effects analyses (DerSimonian and Laird 1986). In addition, because the *Q*-test has low statistical power with few studies (Hardy and Thompson 1998), we also used the *I*^2^ to assess heterogeneity (Higgins and Thompson 2002). Following the thresholds provided by Deeks et al. (2008), we interpreted an *I*^2^ > 30% as reflecting at least moderate heterogeneity. Therefore, when the *Q*-test was not statistically significant, but the *I*^2^ > 30% we also used a random effects model. Using this approach we observed significant heterogeneity across study populations for the PCB-153 association (*p* = 0.01) and therefore we used a random effects model for all analyses. Summary estimates were weighted by the inverse variance of each cohort. To determine the influence of any particular population, we repeated the meta-analyses leaving one population out at the time.

## Results

The median birth weight ranged from 3,210 to 3,750 g, and median gestational age from 38 to 40 weeks. The proportion of cesarean sections was below 25% in all populations except RHEA (43.3%) [see Supplemental Material, Table 1 (http://dx.doi.org/10.1289/ehp.1103767)]. In five populations, > 20% of mothers were < 25 years of age at delivery and in two populations > 20% were > 35 years of age. The proportion of nulliparous women exceeded 40% in nine of the populations.

The median concentration of PCB-153 in cord serum in the 15 study populations ranged from 20 to 484 ng/L [Table 2; also see Supplemental Material, Figure 1 (http://dx.doi.org/10.1289/ehp.1103767)]. The median cord serum PCB-153 concentration in the Faroese population was 3.4 times higher than the overall median of 140 ng/L. The populations with the lowest median PCB-153 concentrations in cord serum (e.g. Warsaw, Kharkiv, HUMIS, and RHEA) also had a narrow range of concentrations compared with the other populations (range from the 10th to the 90th percentiles: 7–83 ng/L compared with 14–1,302 ng/L).

**Figure 1:**
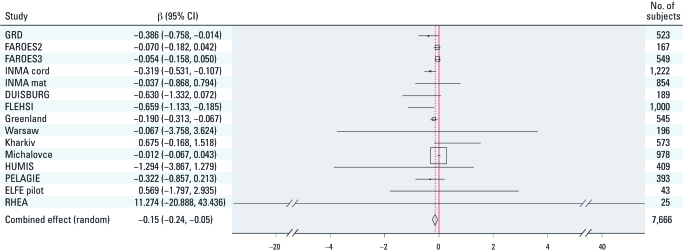
Adjusted regression coefficients (95% CI) of cord serum PCB-153 (ng/L) with birth weight (g). The squares are proportional to the inverse variance of the effect estimation of each cohort. Covariates included in the regression model: child’s gestational age and sex, mother’s region, maternal BMI, height, smoking status during pregnancy, socioeconomic status, mother’s age, parity, and ethnicity, and time of sampling. Greenland, Warsaw, and Kharkiv are part of the INUENDO cohort.

The median level of *p*,*p*´-DDE in cord serum in the 15 populations ranged from 50 to 1,208 ng/L [Table 2; also see Supplemental Material, Figure 2 (http://dx.doi.org/10.1289/ehp.1103767)]. The median cord serum *p*,*p*´-DDE concentration in the Faroese population was 2.2 times higher than the overall median of 528 ng/L.

**Figure 2:**
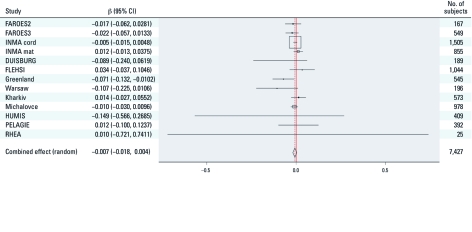
Adjusted regression coefficients (95% CI) of cord serum *p*,*p*´-DDE (ng/L) with birth weight (g). The squares are proportional to the inverse variance of the effect estimation of each cohort. For model covariates, see Figure 1. Greenland, Warsaw, and Kharkiv are part of the INUENDO cohort.

Gestational ages ranged from 25–44 weeks [see Supplemental Material, Table 1 (http://dx.doi.org/10.1289/ehp.1103767)]. Associations between POC concentrations and gestational age, including preterm births, were not statistically significant for any of the individual populations (data not shown) or in the meta-analysis [for PCB-153: β = 6.2 × 10^–6^; 95% confidence interval (CI): –2.5 × 10^–5^, 3.7 × 10^–5^, *p* = 0.18; for *p*,*p*´-DDE: β = –1.2 × 10^–5^; 95% CI: –4.8 × 10^–5^, 2.5 × 10^–5^), *p* = 0.40].

There was a statistically significant inverse association between PCB-153 concentrations in cord serum and birth weight in four populations (GRD, INMA cord, FLEHSI, and Greenland) (Figure 1).

The meta-analysis showed a statistically significant inverse association between PCB-153 concentrations in cord serum and birth weight, corresponding to a 150-g (95% CI: –240, –50 g) reduction per 1-µg/L increase in cord serum PCB-153 (Figure 1). A sensitivity analysis restricted to 3,856 nulliparous women produced a combined estimate of similar magnitude (β = –152 g/µg PCB-153; 95% CI: –341, 37, *p* = 0.12 [see Supplemental Material, Table 3 (http://dx.doi.org/10.1289/ehp.1103767)]. Analyses leaving out one population at the time did not show qualitatively different results from those reported (data not shown). In the meta-regression, biological sample matrix (cord serum, maternal blood/serum, or milk), time period (1990–1999/2000–2008), geographic area (FAROES2, FAROES3, Greenland, and HUMIS vs. other cohorts), and fish consumption (FAROES2, FAROES3, and Greenland) versus other cohorts were not significant predictors of heterogeneity among the study populations (data not shown). Separate meta-analytic regressions by sample matrix were consistent with the overall combined estimate for maternal serum (β = –120 g/µg PCB-143; 95% CI: –260, 10), stronger for the combined estimate for cord serum (β = –290 g/µg PCB-143; 95% CI: –540, –40) and weaker for breast milk (β = –50 g/µg PCB-143; 95% CI: –160, 50).

The meta-analysis of the relation between cord serum *p*,*p*´-DDE and birth weight (Figure 2) did not indicate a statistically significant association (7-g reduction per 1-µg/L increase in cord serum *p*,*p*´-DDE; 95% CI: –18, 4). Analyses leaving one cohort out at a time did not show qualitatively different results. Separate meta-analytic regressions by sample matrix were consistent with the overall result and did not show an association between *p*,*p*´-DDE and birth weight (data not shown).

Associations of birth weight with PCB-153 and *p*,*p*´-DDE were not modified by sex or smoking (data not shown). Associations were also comparable when lipid-adjusted POC concentrations were modeled and when exposures were modeled using quadratic and log-linear transformation (data not shown). The expected associations between birth weight and sex (higher birth weight for males compared to females), tobacco smoking (smoking during pregnancy leading to lower birth weight) and body mass index (mothers with a high BMI have babies with higher birth weights) were demonstrated in all cohorts (data not shown). A sensitivity analysis restricted to infants born at term (e.g., gestational age between 37 and 42 weeks) showed no material changes in effect estimates (data not shown). When adjusting for *p*,*p*´-DDE in the models, the inverse association between PCB-153 and birth weight was not altered, and no significant interaction was found between PCB-153 and *p*,*p*´-DDE on birth weight within the cohorts (data not shown).

## Discussion

In our meta-analysis of 12 European birth cohorts (15 study populations) we observed decreases in birth weight independent of gestational age with increasing fetal PCB-153 concentrations. Across all cohorts birth weight declined by 150 g in association with a 1-µg/L increase of PCB-153 in cord serum—an exposure contrast that is close to the range of exposure levels across the cohorts. In magnitude, this association is comparable to the association between birth weight and smoking about 10 cigarettes per day during pregnancy, and more pronounced than the association with exposure to environmental tobacco smoking, which, according to a review of several earlier studies and meta-analyses, confers an average reduction in birth weight of 30–40 g (Lindbohm et al. 2002). If the observed associations are causal, effects attributable to PCB exposure may be clinically relevant in the studied populations.

In contrast, we found no indications that fetal exposure to the DDT metabolite *p*,*p*´-DDE was related to birth weight, which is consistent with results of a large study based on the U.S. National Collaborative Perinatal Project (CPP) (Longnecker et al. 2001) that examined children born 1959–1966, before DDT was banned in the United States. Although results of the CPP study strongly suggested that DDT exposure was related to preterm birth and small for gestational age babies, no association was detected with maternal serum concentrations < 10 µg/L (corresponding to a concentration of 2.5 µg/L in cord serum), which is higher than DDE concentrations in > 95% of pregnant women participating in our study [see Supplemental Material, Figure 2 (http://dx.doi.org/10.1289/ehp.1103767)].

Fish is an important source of PCB exposure in communities with a high seafood intake (Thorsdottir et al. 2004), but fish meals also contribute substantially to internal PCB levels in other populations (Halldorsson et al. 2008). Fatty fish contains n-3 polyunsaturated fatty acids (PUFA) that in a randomized controlled trial have been shown to prolong gestation and increase birth weight (Olsen et al. 2007). A study of a representative sample of Danish pregnant women that explicitly addressed competitive effects of n-3 polyunsaturated fatty acids and organochlorine contaminants in seafood did not indicate that the effects on birth weight of the PUFA outweighed possible deleterious effects of PCBs or other contaminant exposures in the study population (Halldorsson et al. 2008). Because the balance of effects is determined by the relative exposure levels, these findings may not apply to populations consuming other types of seafood. Other studies have not observed associations between PCBs and birth weight after adjusting for marine n-3 fatty acids (Grandjean et al. 2001). Accurate information on fish intake was not available in all cohorts. In general, we would expect residual confounding due to nondifferential misclassification of fish consumption to bias the association between PCBs and birth weight upward. Therefore, it seems unlikely that misclassification would cause the inverse association that we observed. In addition, fish consumption did not appear to contribute to heterogeneity across the cohorts. It is possible, however, that the misclassification might have been related to other sources of error and possibly to exposure, given that it varied among the cohorts such that any resulting bias might differ from expectations.

The PCB congener mixture in human tissues depends on the source of exposure and the time of sampling relative to exposure (Faroon and Olson 2000). Because PCB congeners vary in toxicity, differences in PCB congener profiles across populations may result in differences in effects on birth weight (if any) and explain some of the heterogeneity of the effect estimates. Although PCB-153 was highly correlated with the non-dioxin-like PCB-138 and PCB-180 in all cohorts (*r*-values ranging from 0.79 to 0.99), the association with the dioxin-like PCB-118 was weaker [*r*-values ranging from 0.23 to 0.83 except in the Faroese [*r* = 0.94 for FAROES2 and 0.90 for FAROES3, respectively (Grimvall et al. 1997)]. Other biopersistent organochlorines that are correlated with PCB-153, such as dioxins, hexachlorobenzene (Eggesbo et al. 2009), hexachlorocyclohexane, dieldrin, and others (Longnecker et al. 2005) may add to the observed heterogeneity. It is not possible to examine possible differential effects of different mixtures of PCB congeners with the available data, but geographic area and calendar time of the study did not contribute to the observed heterogeneity.

Measurements from cord blood reflect exposure at delivery but may not be a good marker of exposure in early pregnancy (Murphy et al. 2010), and maternal blood samples taken in late pregnancy (most samples in this study) may not truly reflect fetal exposure during embryogenesis. In a longitudinal study, Bloom et al. (2007) observed substantially lower serum concentrations of PCB congeners 6 weeks after delivery and in early pregnancy compared with the period before conception. The magnitude of change from early to late pregnancy (if any) is, to the best of our knowledge, unknown. Extrapolating fetal exposure from breast milk samples is less reliable, because exposure assessment is retrospective and exposure levels decline with time after delivery because of elimination through breast milk (Redding et al. 2008). On the other hand, results of a recent study suggest that while variable, the average monthly decrease in milk PCB is only 1% (Hooper et al. 2007). Thus, the error in exposure introduced by using concentrations in milk one month after birth as a surrogate for prenatal exposure may be limited. We were not able to fully address the effects of different sampling times relative to pregnancy; but a poor proxy of a causal agent is more likely to result in dilution of risk estimates and therefore in particular is an issue in studies with no associations.

The conversion of concentrations measured from maternal serum and breast milk into those of cord serum was for descriptive purposes only, whereas the meta-analysis was based upon within-population regression estimates of exposures that were measured in a single biological matrix. We did not attempt to perform exposure–response analyses across the various populations, and thus did not include the absolute values of exposures in the meta-analysis.

The specificity of results indicating associations of birth weight with PCBs but not *p*,*p*´-DDE, as well as demonstration of the expected associations between birth weight and, respectively, infant sex and maternal tobacco smoking and BMI, supports the validity of findings and suggests that the association between PCB-153 and birth weight was not merely an artifact of study design or analysis. The correlation between concentrations of PCB-153 and *p*,*p*´-DDE was about 0.6 in most cohorts, but adjustment by *p*,*p*´-DDE in the individual cohorts did not attenuate the inverse association between PCB and birth weight, and there was no significant interaction between PCB-153 and *p*,*p*´-DDE on birth weight within or across cohorts.

Are results biased by reverse causation? Large maternal weight gain is correlated with high birth weight and could reduce POC levels because of dilution, thus producing an artificially negative association between POC concentration and birth weight. The much stronger association of PCB-153 than *p*,*p*´-DDE with birth weight is inconsistent with the dilution hypothesis, unless partitioning of PCBs into adipose tissue is greater than for DDE. But available data do not indicate that placental transfer of DDE deviates from PCBs in average (Needham et al. 2011). Moreover, in cohorts where data on weight gain during pregnancy were available [INMA and PELAGIE (Perturbateurs endocriniens: Étude Longitudinale sur les Anomalies de la Grossesse, l’Infertilité et l’Enfance)], additional analyses adjusting for weight gain during pregnancy did not change the risk estimates materially. For instance, for the INMA cord subcohort, the weight gain unadjusted risk estimate was –0.30 (95% CI: –0.57, –0.04) and the weight gain adjusted estimate was –0.31 (95% CI: –0.57, –0.05).

The discrepant findings in several earlier studies are puzzling (Dar et al. 1992; Fein et al. 1984; Gladen et al. 2003; Grandjean et al. 2001; Karmaus and Zhu 2004; Longnecker et al. 2005; Mendez et al. 2010; Murphy et al. 2010; Patandin et al. 1998; Rylander et al. 1995, 1996, 1998; Sonneborn et al. 2008; Vartiainen et al. 1998; Wojtyniak et al. 2010). Among the populations included in this meta-analysis, only four showed a significant inverse association between PCB-153 and birth weight, of which one to two might be expected as chance findings at the 5% significance level (15 populations and 2 compounds = 30 comparisons). Reasons may be inadequate sample sizes, exposure misclassification, or exposure profile heterogeneity. However, we acknowledge that more consistent findings in earlier studies and across populations in this study would add to the credibility of the overall result.

Although the possible mode of action for effects of PCBs on birth weight is uncertain, the endocrine-disrupting properties of PCBs could be involved. Estrogens play a pivotal role in promoting fetal growth (Kaijser et al. 2000). Both estrogenic and anti-estrogenic activity has been observed for non-dioxin-like PCBs (NDL-PCBs). A recent study analyzing the toxicity profiles of 24 PCB congeners by *in vitro* assays revealed that higher chlorinated NDL-PCBs were weak estrogen receptor (ER) antagonists, and that several NDL-PCBs inhibited estradiol-sulfotransferase activity (Hamers et al. 2011). Antiestrogenic congeners may inhibit fetal growth by their intrinsic activity or by disruption of endogenous estrogen metabolism. In addition, both estrogenic and antiestrogenic activity has been observed for hydroxylated metabolites of lower chlorinated non-dioxin-like PCBs (Connor et al. 1997), which are transferred more efficiently through placenta than the parent compounds (Park et al. 2008). PCBs exhibit also a weak antiandrogenic activity, which may also have an impact on fetal growth (Luke et al. 2005). Finally, PCBs are also known to interfere with thyroid hormone status in animals and humans [for reviews see Klaassen and Hood (2001) and Brouwer et al. (1998)]. *In vitro* assays showed that several NDL-PCBs inhibit estradiol-sulfotransferase activity and bound to transthyretin (Hamers et al. 2011). It is well known that maternal hypothyroidism is related to low birth weight (Blazer et al. 2003).

Low birth weight represents a mix of preterm delivery and reduced fetal growth. Because PCB levels were not associated with duration of gestation in either sex, our results suggest that PCB dose may be related to birth weight restriction rather than preterm delivery. This hypothesis was corroborated by an analysis restricted to infants born at term that showed no material changes in effect estimates, consistent with several recent studies (Halldorsson et al. 2008; Hertz-Picciotto et al. 2005; Longnecker et al. 2001; Sonneborn et al. 2008).

In two studies, reduced birth weight has been observed in association with PCBs among boys only (Hertz-Picciotto et al. 2005; Sonneborn et al. 2008). This observation was not corroborated by our analyses. Earlier reports on sex-specific effects are not supported by animal or mechanistic evidence and may represent chance findings.

## Conclusion

Our harmonized meta-analysis of 12 European mother–child cohorts including > 7,000 pregnancies suggests that low-level PCB exposure (or correlated exposures) impairs fetal growth, whereas current levels of *p*,*p*´-DDE were not associated with birth weight adjusted for gestational age. On average, birth weight declined by 150 g per 1-µg/L increase in PCB-153 cord serum concentration. The study adds to the mounting evidence that low-level exposure to PCBs is inversely associated with fetal growth.

## Supplemental Material

(344 KB) PDF
