# Supplementary material for: Birth Weight and Prenatal Exposure to Polychlorinated Biphenyls (PCBs) and Dichlorodiphenyldichloroethylene (DDE): A Meta-analysis within 12 European Birth Cohorts
Source: Environ Health Perspect. 2011 Oct 13;120(2):162–70. doi: 10.1289/ehp.1103767 (PMC3279442; doi:10.1289/ehp.1103767)
Supplement: (344 KB) PDF [file ehp.1103767.s001.pdf]

Supplemental Material

Prenatal Exposure to Polychlorinated Biphenyls (PCB) and Dichlorodiphenyldichloroethylene (DDE) and Birth Weight: a Meta-analysis within 12 European Birth Cohorts

Eva Govarts, Mark Nieuwenhuijsen, Greet Schoeters, Ferran Ballester, Karolien Bloemen, Michiel de Boer, Cécile Chevrier, Merete Eggesbø, Monica Guxens, Ursula Krämer, Juliette Legler, David Martínez, Lubica Palkovicova, Evridiki Patelarou, Ulrich Ranft, Arja Rautio, Maria Skaalum Petersen, Remy Slama, Hein Stigum, Gunnar Toft, Tomas Trnovec, Stéphanie Vandentorren, Pál Weihe, Nynke Weisglas Kuperus, Michael Wilhelm, Jürgen Wittsiepe, and Jens Peter Bonde and OBELIX/ENRIECO

Content

Conversion of maternal serum and breast milk levels to cord serum levels, p 2-3.

Supplemental Material, Table 1, Cohort characteristics of the ENRIECO/OBELIX birth cohorts with biological PCB 153/p,p'-DDE exposure biomarkers, p 4-5.

Supplemental Material, Table2, Chemical-analytical methods and detection/quantification limits of the ENRIECO/OBELIX birth cohorts, p 6.

Supplemental Material, Table 3, Adjusted regression coefficient of exposure biomarkers PCB 153 and p,p'-DDE (ng/L) with birth weight (grams) estimated separately for each ENRIECO/OBELIX birth cohort and by meta-analysis (random effects model). Nulliparous, p 7.

Supplemental Material, Figure 1, Range of PCB 153 concentration in cord serum (P10, median, P90) (ng/L), using actual observed and estimated concentrations, of the ENRIECO/OBELIX birth cohorts, p 8.

Supplemental Material, Figure 2, Range of p,p'-DDE concentration in cord serum (P10, median, P90) (ng/L), using actual observed and estimated concentrations, of the ENRIECO/OBELIX, p 9.

References, p 10.

Conversion of maternal serum and breast milk levels to cord serum levels

To obtain the same exposure measure of PCB 153 and p,p'-DDE in all the cohorts, concentrations in maternal milk (FAROES3, HUMIS, and ELFE pilot cohorts), maternal serum (FAROES2, INMA, INUENDO, and RHEA cohorts) and maternal whole blood (DUISBURG) needed to be converted to wet weight cord serum levels. Needham et al. (Needham et al. 2011) provided data on the average ratio of organohalogen concentrations in cord serum and human milk in comparison with the concentrations in maternal serum, with and without lipid adjustment. The average wet weight cord serum/maternal serum ratio was 0.20 and the lipid-based average breast milk/maternal serum concentration was estimated to 1.48. Because the mean lipid concentration of maternal serum for this estimation amounted to 8.9 g/L, the ratio of organohalogen concentration in human milk fat versus wet weight maternal serum concentration was 0.166 L/g lipid (= 1.48 / 8.9 g lipid/L). As such, the ratio of concentration in human milk fat to that in wet weight maternal serum was estimated to be 6 (=1/0.166). Therefore, a conversion factor for lipid adjusted breast milk levels to wet weight cord serum levels of 1.2 g lipid/L (= 6 g lipid/L * 0.2) was assumed. To convert the maternal whole blood wet weight measurements of the DUISBURG cohort to wet weight serum concentrations a conversion factor of 1.8 was used which has been estimated by Jotaki et al. (Jotaki et al. 2011) for PCB congeners. In summary, the following conversion formulas were used for PCB 153 and p,p'-DDE levels in two different matrices:

[1] cord serum level (ng/L) = 0.20 * maternal serum level (ng/L)

[2] cord serum level (ng/L) = 1.20 * breast milk level (ng/g fat)

[3] cord serum level (ng/L) = 0.36 * maternal whole blood level (ng/L)

Those ENRIECO cohortswith toxicant analyses available from at least two matrices provided the possibility to check consistency between the conversion factors found in the literature (Jotaki et al. 2011;Needham et al. 2011) and those obtained from ENRIECO data. From the INMA Sabadell cohort dataset we obtained an average ratio of PCB 153 concentration in human milk fat versus wet weight maternal serum of 0.188 L/g lipid (based on 251 samples). The corresponding ratio in the DUISBURG

cohort was 0.132 L/g lipid (based on 147 samples) where the conversion factor of 1.8 (Jotaki et al. 2011) for the whole blood measurements was assumed. Both the INMA and the DUISBURG estimates correspond closely to the ratio of 0.166 L/g lipid derived from the Needham et al. data (Needham et al. 2011). In a similar way as for PCB 153, average ratios of 0.072 L/g lipid (INMA cohort) and 0.115 L/g lipid (DUISBURG cohort) for p,p'-DDE concentrations in lipid adjusted maternal milk versus wet weight maternal serum p,p'-DDE levels were found. Furthermore, in the MICHALOVCE cohort a conversion factor of 0.2 (based on 1025 samples) was obtained for converting PCB 153 wet weight concentrations in maternal serum to those in cord serum. For p,p'-DDE a conversion factor of 0.23 (based on 1018 samples) was obtained. Again, these ENRIECO derived conversion factors are very close to the ratios provided by Needham et al. (Needham et al. 2011).

Supplemental Material, Table 1: Cohort characteristics of the ENRIECO/OBELIX birth cohorts with biological PCB 153/p,p'-DDE exposure biomarkers.

Characteristics	GRD	FAROES2	FAROES3	INMA cord	INMA mat	Duisburg	FLEHSI	Greenland	Warsaw	Kharkiv	Michalovce	HUMIS	PELAGIE	ELFE pilot	RHE
N	523	167	549	1227	856	189	1015	546	199	577	1036	409	396	43	30
Birth weight (g)	3500 (2140-5000)	3650 (2500-4800)	3750 (2250-5500)	3250 (1200-4880)	3290 (770-4785)	3520 (2200-4925)	3390 (1245-5575)	3593 (845-5300)	3490 (610-5140)	3300 (1140-4700)	3350 (950-5060)	3670 (1865-5100)	3370 (1070-4760)	3340 (2700-4210)	3210 (214 3920
Gestational Age (GA) (weeks)	40 (37-43)	40 (36-42)	40 (34-42)	39.9 (29.7-42.6)	39.9 (28.1-42.3)	40 (35-43)	39 (31-42)	40 (25-44)	40 (25-42)	39 (29-42)	40 (30-43)	40.43 (35-44)	40 (27-42)	40 (36-41)	38 (35-4
Determination of GA															
1: 1st day last menstruation	/	/	/	382 (31.1)	/	/	/	/	/	/	1036 (100)	/	/	/	/
2: Ultrasound	/	167 (100)	549 (100)	/	/	/	/	/	/	/	/	/	396 (100)	/	/
3: Combination 1&2	/	/	/	845 (68.9)	856 (100)	189 (100)	/	546 (100)	199 (100)	577 (100)	/	409 (100)	/	43 (100)	30 (1
4: Unknown	523 (100)	/	/	/	/	/	1015 (100)	/	/	/	/	/	/	/	/
Term															
1: preterm (<37 weeks)	0	3 (1.8)	9 (1.6)	52 (4.2)	29 (3.4)	0	35 (3.4)	28 (5.1)	12 (6.0)	11 (1.9)	25 (2.4)	15 (3.7)	10 (2.5)	1 (2.3)	7 (23
2: term (37-42 weeks)	520 (99.4)	164 (98.2)	540 (98.4)	1157 (94.3)	818 (95.6)	187 (98.9)	980 (96.6)	510 (93.4)	187 (94.0)	566 (98.1)	1009 (97.4)	391 (95.6)	386 (97.5)	42 (97.7)	23 (7
3: over term (>42 weeks)	3 (0.6)	0	0	18 (1.5)	9 (1.1)	2 (1.1)	0	8 (1.5)	0	0	2 (0.2)	3 (0.7)	0	0	0
Region[a]		/	/			/		/	/	/			/	/	/
1:	191 (36.5)			382 (31.1)	/		193 (19.0)				753 (72.7)	54 (13.2)			
2:	191 (36.5)			/	/		132 (13.0)				283 (27.3)	64 (15.6)			
3:	141 (27.0)			498 (40.6)	/		189 (18.6)					77 (18.8)			
4:				/	589 (68.8)		115 (11.3)					105 (25.7)			
5:				24 (2.0)	/		123 (12.1)					109 (26.7)			
6:				323 (26.3)	267 (31.2)		21 (2.1)								
7:							183 (18.0)								
8:							59 (5.8)								
Child gender															
Boy	288 (55.1)	83 (49.7)	292 (53.2)	651 (53.1)	423 (49.4)	89 (47.1)	531 (52.3)	295 (54.0)	97 (48.7)	304 (52.7)	529 (51.1)	209 (51.1)	200 (50.5)	22 (51.2)	21 (7
Girl	235 (44.9)	84 (50.3)	257 (46.8)	576 (46.9)	433 (50.6)	100 (52.9)	484 (47.7)	251 (46.0)	102 (51.3)	273 (47.3)	507 (48.9)	200 (48.9)	196 (49.5)	21 (48.8)	9 (30
Maternal age at delivery															
1: <25 years	61 (11.7)	44 (26.3)	126 (23.0)	102 (8.3)	35 (4.1)	22 (11.6)	141 (13.9)	253 (46.3)	13 (6.5)	307 (53.2)	480 (46.3)	68 (16.6)	33 (8.3)	1 (2.3)	5 (16
2: 25-29 years	227 (43.4)	60 (35.9)	156 (28.4)	373 (30.4)	233 (27.2)	40 (21.2)	395 (38.9)	122 (22.3)	121 (60.8)	169 (29.3)	340 (32.8)	162 (39.6)	150 (37.9)	13 (30.2)	6 (20
3: 30-34 years	190 (36.3)	42(25.1)	192 (35.0)	517 (42.1)	392 (45.8)	81 (42.9)	376 (37.0)	86 (15.8)	56 (28.1)	81 (14.0)	170 (16.4)	121 (29.6)	152 (38.4)	21 (48.8)	18 (6
4: 35+ years	45 (8.6)	21 (12.6)	75 (13.7)	235 (19.2)	196 (22.9)	46 (24.3)	103 (10.1)	85 (15.6)	9 (4.5)	20 (3.5)	46 (4.4)	58 (14.2)	61 (15.4)	8 (18.6)	1 (3.
Maternal BMI															
1: < 18.5 kg/m²	27 (5.2)	6 (3.6)	23 (4.2)	56 (4.6)	43 (5.0)	4 (2.1)	56 (5.5)	17 (3.1)	11 (5.5)	81 (14.0)	127 (12.3)	13 (3.2)	30 (7.6)	2 (4.7)	3 (10
2: 18.5-24 kg/m²	365 (69.8)	121 (72.5)	360 (65.6)	876 (71.4)	594 (69.4)	134 (70.9)	684 (67.4)	331 (60.6)	168 (84.4)	419 (72.6)	683 (65.9)	257 (62.8)	304 (76.8)	32 (74.4)	20 (6
3: 25-29 kg/m²	94 (18.0)	33 (19.8)	127 (23.1)	209 (17.0)	158 (18.5)	30 (15.9)	189 (18.6)	141 (25.8)	13 (6.5)	63 (10.9)	126 (12.2)	96 (23.5)	44 (11.1)	9 (20.9)	6 (20
4: 30+ kg/m²	37 (7.1)	7 (4.2)	39 (7.1)	81 (6.6)	61 (7.1)	21 (11.1)	71 (7.0)	56 (10.3)	4 (2.0)	10 (1.7)	54 (5.2)	43 (10.5)	15 (3.8)	0	1 (3.
Maternal height (cm)	170 (150-193)	163 (150-183)	165 (148-184)	162 (135-185)	163 (145-180)	167 (151-183)	167 (150-184)	162 (145-180)	167 (150-182)	165 (150-181)	165 (133-186)	168 (149-199)	164 (146-190)	164 (148-178)	162.5 (152-
Parity															
0	259 (49.5)	47 (28.1)	153 (27.9)	641 (52.2)	480 (56.1)	86 (45.5)	613 (60.4)	175 (32.1)	182 (91.5)	458 (79.4)	437 (42.2)	163 (39.9)	172 (43.4)	14 (32.6)	2 (6.
1	246 (47.0)	50(29.9)	184 (33.5)	465 (37.9)	320 (37.4)	63 (33.3)	275 (27.1)	162 (29.7)	15 (7.5)	104 (18.0)	345 (33.3)	165 (40.3)	143 (36.1)	16 (37.2)	12 (4
2+	18 (3.4)	70 (41.9)	212 (38.6)	121 (9.9)	54 (6.3)	40 (21.2)	127 (12.5)	209 (38.3)	2 (1.0)	15 (2.6)	251 (24.2)	81 (19.8)	81 (20.5)	13 (30.2)	11 (3
Socioeconomic status (SES)[a]															
1:	66 (12.6)	27 (16.2)	31 (5.6)	453 (36.9)	198 (23.1)	9 (4.8)	15 (1.5)	294 (53.8)	32 (16.1)	309 (53.6)	216 (20.8)	53 (13.0)	73 (18.4)	13 (30.2)	6 (20
2:	163 (31.2)	43(25.7)	132 (24.0)	434 (35.4)	342 (40.0)	72 (38.1)	103 (10.1)	70 (12.8)	1 (0.5)	198 (34.3)	262 (25.3)	83 (20.3)	69 (17.4)	30 (69.8)	18 (6

3:	294 (56.2)	97 (58.1)	386 (70.3)	340 (27.7)	316 (36.9)	45 (23.8)	676 (66.6)	174 (31.9)	17 (8.5)	15 (2.6)	480 (46.3)	183 (44.7)	254 (64.1)	/	6 (20
4:	/	/	/	/	/	63 (33.3)	221 (21.8)	8 (1.5)	149 (74.9)	55 (9.5)	78 (7.5)	90 (22.0)	/	/	/
Smoking during pregnancy[b]															
1: not smoking	387 (74.0)	113 (67.7)	397 (72.3)	860 (70.1)	623 (72.8)	146 (77.2)	849 (83.6)	56 (10.3)	161 (80.9)	446 (77.3)	879 (84.8)	364 (89.0)	339 (85.6)	43 (100)	23 (7
2: 1-9 cigarettes/day	136 (26.0)	36 (21.6)	115 (20.9)	268 (21.8)	172 (20.1)	26 (13.8)	102 (10.0)	335 (61.4)	21 (10.6)	84 (14.6)	157 (15.2)	26 (6.4)	42 (10.6)	0	3 (10
3: 10+ cigarettes/day	0	18 (10.8)	37 (6.7)	99 (8.1)	61 (7.1)	17 (9.0)	64 (6.3)	155 (28.4)	17 (8.5)	47 (8.1)	/	19 (4.6)	15 (3.8)	0	4 (13
Drinking during pregnancy															
1: no alcoholic drinks	365 (69.8)	145 (86.8)	515 (93.8)	997 (81.3)	684 (79.9)	168 (88.9)	927 (91.3)	420 (76.9)	66 (33.2)	364 (63.1)	863 (83.3)	369 (90.2)	340 (85.9)	43 (100)	22 (7
2: 1-9 alcoholic drinks/week	158 (30.2)	22 (13.2)	34 (6.2)	230 (18.7)	171 (20.0)	21 (11.1)	84 (8.3)	76 (13.9)	128 (64.3)	212 (36.7)	173 (16.7)	40 (9.8)	46 (11.6)	0	1 (3.[illegible]
3: 10+ alcoholic drinks/week	0	0	0	0	1 (0.1)	0	4 (0.4)	50 (9.2)	5 (2.5)	1 (0.2)	/	0	10 (2.5)	0	7 (23
Ethnicity															
1: Caucasian	523 (100)	160 (95.8)	538 (98.0)	1180 (96.2)	830 (97.0)	189 (100)	/	0	199 (100)	577 (100)	800 (77.2)	366 (89.5)	396 (100)	/	/
2: Inuit	/	/	/	/	/	/	/	546 (100)	0	0	/	/	0	/	/
3: Roma	/	/	/	/	/	/	/	0	0	0	220 (21.2)	/	0	/	/
4: Other	/	/	/	47 (3.8)	26 (3.0)	/	/	0	0	0	/	41 (10.0)	0	/	/
9: Unknown	/	7 (4.2)	11 (2.0)	/	/	/	1015 (100)	0	0	0	16 (1.5)	/	0	43 (100)	30 (1
Time sample collection															
1: First trimester	0	0	0	0	545 (63.7)	0	0	41 (7.5)	4 (2.0)	162 (28.1)	0	0	0	0	0
2: Second trimester	0	0	0	0	309 (36.1)	2 (1.1)	0	127 (23.3)	13 (6.5)	166 (28.8)	0	0	0	0	0
3: Third trimester	0	167 (100)	0	0	2 (0.2)	175 (92.6)	0	84 (15.4)	178 (89.4)	149 (25.8)	0	0	0	0	2 (6.[illegible]
4: Postnatal	523 (100)	0	549 (100)	1227 (100)	0	12 (6.3)	1015 (100)	294 (53.8)	4 (2.0)	100 (17.3)	1036 (100)	409 (100)	396 (100)	43 (100)	28 (9
Caesarian section															
1: Yes	/	20 (12.0)	55 (10.0)	145 (11.8)	101 (11.8)	42 (22.2)	51 (5.0)	/	/	/	/	58 (14.2)	50 (12.6)	1 (2.3)	13 (4
2: No	523 (100)	147 (88.0)	494 (90.0)	968 (78.9)	596 (69.6)	147 (77.8)	964 (95.0)	/	/	/	/	325 (79.5)	339 (85.6)	41 (95.3)	17 (5
9: Unknown	/	/	/	114 (9.3)	159 (18.6)	/	/	546 (100)	199 (100)	577 (100)	1036 (100)	26 (6.4)	7 (1.8)	1 (2.3)	/

Continuous measures described by median (min-max); categorical measures described by frequencies (%).

[a] Cohort-specific categories were allowed for region and socioeconomic status.

[b] In the PELAGIE cohort smoking status at inclusion used as proxy of smoking during pregnancy.

Supplemental Material, Table 2: Chemical-analytical methods and detection/quantification limits of the ENRIECO/OBELIX birth cohorts

Cohort	Matrix	Extraction (phase)	Gas chromatograph separation	Detector type	Method of lipid analysis	LOD/LOQ PCB 153	LOD/LOQ p,p'-DDE
**Groningen Rotterdam**	Cord plasma	Liquid-liquid	High resolution	ECD	ND	10 ng/L	/
**Dusseldorf**	Cord serum	Liquid -liquid	High resolution	ECD	ND	10 ng/L	/
**FAROES2**	Maternal serum	Solid	High resolution	ECD	Gravimetric	80 ng/L	80 ng/L
**FAROES3**	Breast milk	Solid-liquid	High resolution	ECD	Gravimetric	5 ng/g fat	3 ng/g fat
**INMA cord**	Cord serum	Liquid-liquid	Low resolution	ECD & MS	Enzymatic	32.4-71 ng/L	19-500 ng/L
**INMA mat**	Maternal serum	Liquid-liquid	Low resolution	ECD & MS	Enzymatic	14.2 ng/L	71 ng/L
**DUISBURG**	Maternal blood	Liquid-liquid	High resolution	MS	Gravimetric	5 ng/L	5 ng/L
**FLEHSI**	Cord plasma	Solid	Low resolution	MS	Gravimetric/ Enzymatic	20 ng/L	20 ng/L
**INUENDO**	Maternal serum	Solid	High resolution	MS	Enzymatic	50 ng/L	100 ng/L
**Michalovce**	Cord serum	Solid	High resolution	ECD	Enzymatic	3.4-23 ng/L	1.3-13 ng/L
**HUMIS**	Breast milk	Liquid-liquid	High resolution	ECD	Gravimetric	0.458 ng/g fat	0.224 ng/g fat
**PELAGIE**	Cord serum	Solid	High resolution	MS	Enzymatic	10 ng/L	50 ng/L
**ELFE pilot**	Breast milk	Liquid-liquid	High resolution	MS	Gravimetric	0.885 ng/g fat	/
**RHEA**	Maternal serum	Liquid-liquid	High resolution	MS	ND	4 ng/L	5 ng/L

ECD = electron capture detection; MS = mass spectrometry; LOD = limit of detection; LOQ = limit of quantification; ND = not determined.

Supplemental Material, Table 3: Adjusted[a] regression coefficient of exposure biomarkers PCB 153 and p,p'-DDE (ng/L) with birth weight (grams) estimated separately for each ENRIECO/OBELIX birth cohort and by meta-analysis (random effects model). Nulliparous.

	**PCB 153 (ng/L)**			**p,p'-DDE (ng/L)**		
**Cohort**	**N**	***β***	**95% CI**	**N**	***β***	**95% CI**
**GRD**	280	-0.586	-1.121; -0.051	-	-	-
**FAROES2**	47	-0.294	-0.604; 0.016	47	-0.071	-0.173; 0.031
**FAROES3**	153	0.074	-0.142; 0.290	153	0.021	-0.055; 0.097
**INMA cord**	638	-0.164	-0.431; 0.103	779	0.004	-0.010; 0.018
**INMA mat**	480	0.689	-0.397; 1.775	480	0.025	-0.067; 0.117
**DUISBURG**	90	-0.125	-2.220; 1.970	86	0.094	-0.224; 0.412
**FLEHSI**	602	-0.837	-1.472; -0.202	630	-0.020	-0.116; 0.076
**Greenland**	175	-0.255	-0.618; 0.108	175	-0.293	-0.710; 0.124
**Warsaw**	179	0.366	-3.242; 3.974	182	-0.090	-0.206; 0.026
**Kharkiv**	455	0.507	-0.448; 1.462	455	-0.026	-0.073; 0.021
**Michalovce**	424	0.080	-0.000; 0.160	424	-0.001	-0.040; 0.038
**HUMIS**	162	-2.231	-7.151; 2.689	162	-0.085	-1.663; 1.493
**PELAGIE**	171	-0.614	-1.537; 0.309	171	0.026	-0.113; 0.165
**ELFE pilot[b]**	.	.	.	-	-	-
**RHEA[c]**	.	.	.	.	.	.
**Combined**	3,856	-0.152	-0.341; 0.037	3,744	0.000	-0.012; 0.012

[a] The model with birth weight as an outcome was adjusted for child's gestational age and gender, mother's region, maternal BMI, height, smoking status during pregnancy, socioeconomic status, mother's age, parity, ethnicity and time of sampling. [b] only 14 nulliparous cases [c] only 2 nulliparous cases

Supplemental Material, Figure 1: Range of PCB 153 concentration in cord serum (P10, median, P90) (ng/L), using actual observed and estimated concentrations, of the ENRIECO/OBELIX birth cohorts

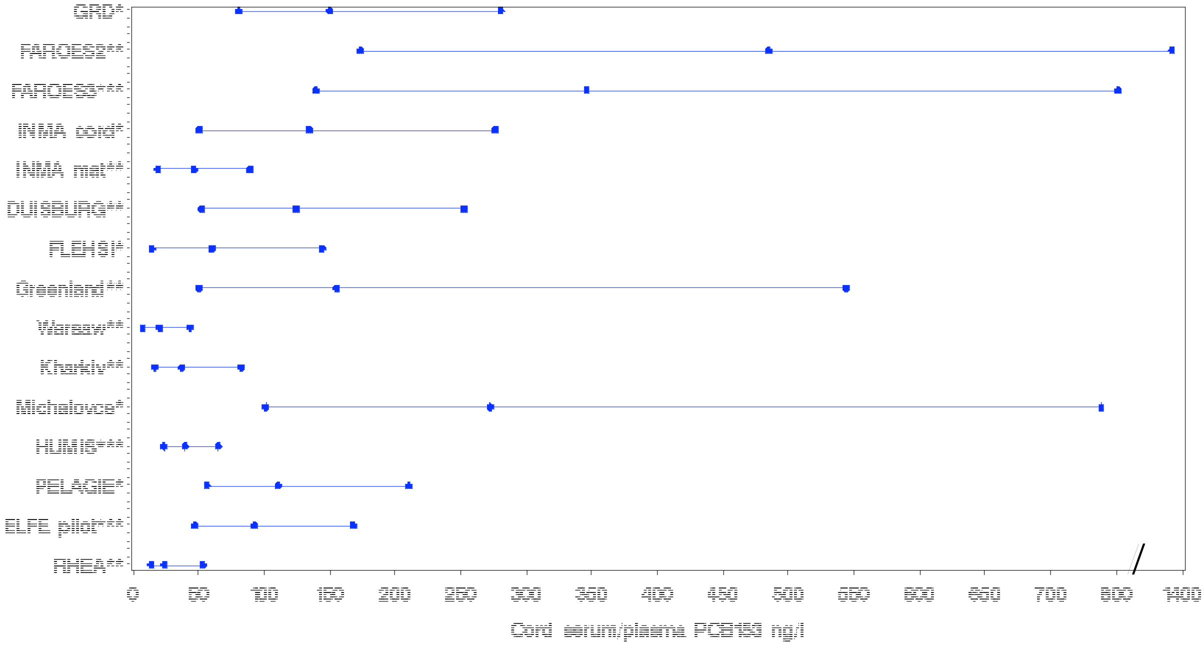

Actual observed* and estimated cord serum concentrations based on measured concentrations in maternal serum (whole blood for Duisburg)** or breast milk*** (see Supplemental Material, pages 2-3 for additional information on conversions).

Supplemental Material, Figure 2: Range of p,p'-DDE concentration in cord serum (P10, median, P90) (ng/L), using actual observed and estimated concentrations, of the ENRIECO/OBELIX birth cohorts

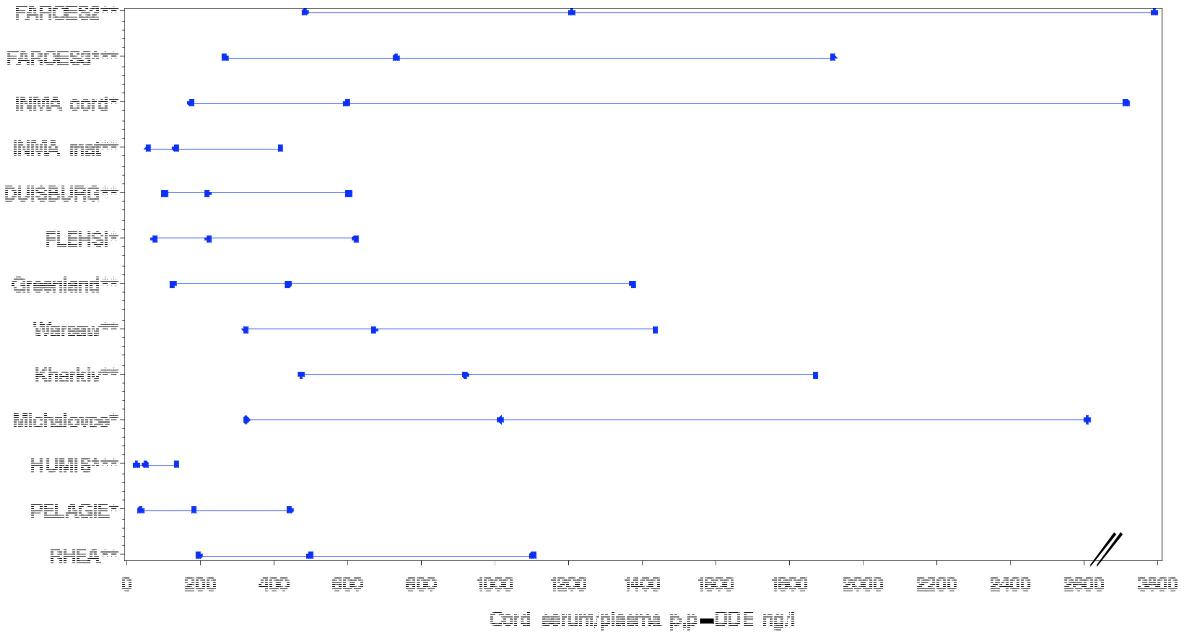

Actual observed* and estimated cord serum concentrations based on measured concentrations in maternal serum (whole blood for Duisburg)** or breast milk*** (see Supplemental Material, pages 2-3 for additional information on conversions).

Reference List

Jotaki T, Fukata H, Mori C. 2011. Confirmation of polychlorinated biphenyl (PCB) distribution in the blood and verification of simple quantitative method for PCBs based on specific congeners. Chemosphere 82: 107-113.

Needham LL, Grandjean P, Heinzow B, Jorgensen PJ, Nielsen F, Patterson DG, et al. 2011. Partition of Environmental Chemicals between Maternal and Fetal Blood and Tissues. Environ Sci Technol 45: 1121-1126.
